# Engineering antioxidant ceria-zirconia nanomedicines for alleviating podocyte injury in rats with adriamycin-induced nephrotic syndrome

**DOI:** 10.1186/s12951-023-02136-2

**Published:** 2023-10-19

**Authors:** Lili Liu, Meiqi Chang, Rong Yang, Li Ding, Yu Chen, Yulin Kang

**Affiliations:** 1 School of Medicine, Anhui University of Science and Technology, Huainan, 232000 People’s Republic of China; 2 Department of Nephrology and Rheumatology, Shanghai Children’s Hospital, School of Medicine, Shanghai Jiao Tong University, Shanghai, 200062 People’s Republic of China; 3 Laboratory Center, Shanghai Municipal Hospital of Traditional Chinese Medicine, Shanghai University of Traditional Chinese Medicine, Shanghai, 200071 People’s Republic of China; 4 Department of Pediatrics, Shanghai Tenth People’s Hospital, School of Medicine, Tongji University, Shanghai, 200072 People’s Republic of China; 5 Department of Medical Ultrasound, National Clinical Research Center of Interventional Medicine, Shanghai Tenth People’s Hospital, Tongji University Cancer Center, Tongji University School of Medicine, Tongji University, Shanghai, 200072 People’s Republic of China; 6 Materdicine Lab, School of Life Sciences, Shanghai University, Shanghai, 200444 People’s Republic of China

**Keywords:** Reactive oxygen species, Ceria-zirconia nanoparticles, Primary nephrotic syndrome, Podocyte

## Abstract

**Background:**

Primary nephrotic syndrome (PNS) is characterized by edema, heavy proteinuria, hypoalbuminemia and hyperlipidemia. Moreover, podocyte injury is the key pathological change of PNS. Even though the pathophysiological etiology of PNS has not been fully understood, the production of excessive reactive oxygen species (ROS) plays an important role in the development and progression of the disease. Glucocorticoids are the first-line medications for patients with PNS, but their clinical use is hampered by dose-dependent side effects. Herein, we accelerated the rate of conversion from Ce^4+^ to Ce^3+^ by doping Zr^4+^ in ceria-zirconia nanomedicines to treat the PNS rat model by removal of ROS.

**Results:**

The engineered Ce_0.7_Zr_0.3_O_2_ (7CZ) nanomedicines significantly improved the ROS scavenging ability of podocytes at a very low dose, enabling effective inhibition of podocyte apoptosis and actin cytoskeleton depolymerization induced by adriamycin (ADR). Accordingly, podocyte injury was effectively alleviated in rat models of ADR-induced nephrotic syndrome, as confirmed by serum tests and renal tissue staining. Moreover, the mRNA sequencing assay revealed the protective molecular signaling pathways of 7CZ nanomedicines in podocytes.

**Conclusion:**

7CZ nanomedicines were highly effective in protecting against ADR-induced podocyte injury in vitro and in vivo at a very low concentration.

**Graphical Abstract:**

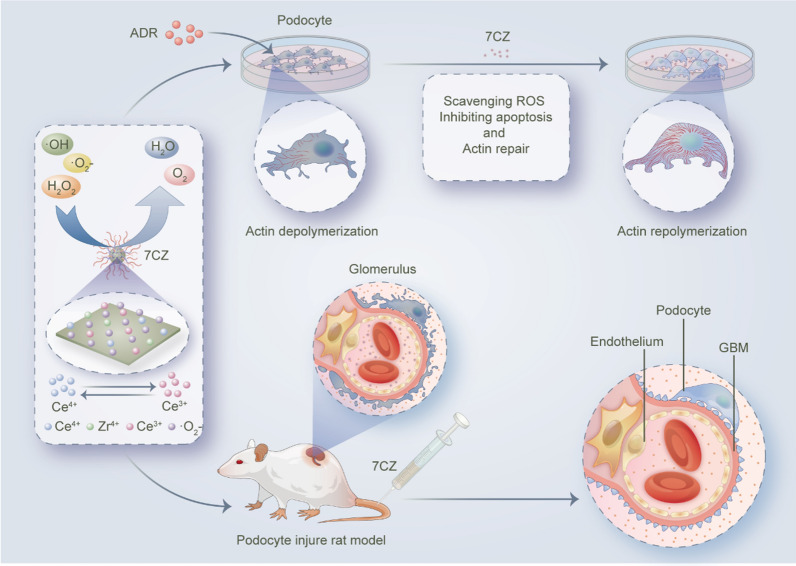

**Supplementary Information:**

The online version contains supplementary material available at 10.1186/s12951-023-02136-2.

## Introduction

Primary nephrotic syndrome (PNS) is commonly manifested as edema, heavy proteinuria, hypoalbuminemia and hyperlipidemia caused by injured glomerular filtration barrier. The incidence of PNS ranges from 1 to 18 per 100,000 children worldwide [1]. Minimal-change nephrotic syndrome (MCNS) is the most common pathological type of PNS in children. The etiology of MCNS is still unclear, even though it may be a T-cell-related disease that results in podocyte dysfunction [2]. Podocytes are polarized epithelial cells that line outside of the glomerular filtration barrier. The impaired podocyte structure or function leads to glomerular filtration dysfunction. Large amounts of proteinuria will accelerate the progression of PNS and eventually may develop into end-stage renal disease [3]. Glucocorticoids are the first-line medications for patients with PNS. However, the frequent relapses of PNS require additional immunosuppressive agents. The risk of adverse events including infection and obesity is greatly increased due to the prolonged treatment with glucocorticoids and immunosuppressive agents [4]. Therefore, the long-term prognosis of patients with difficult-to-treat PNS is not promising due to the lack of efficient therapeutic approaches. Hence, an effective and safe approach is required for patients with PNS.

An appropriate amount of reactive oxygen species (ROS) is essential to keep intracellular signaling transduction, but excessive production of ROS may result in cell damage or apoptosis [5]. Studies have indicated that excessive ROS production is associated with atherosclerosis, diabetes, neurodegenerative diseases, ischemia/reperfusion injury, rheumatoid arthritis, obstructive sleep apnea, pathogenesis of cancer and other diseases. Furthermore, ROS is known to cause damage to podocytes which can lead to a range of nephrotic syndromes (NS) [6–8]. These effects include podocyte apoptosis, rearrangement of the actin cytoskeleton, disruption of the slit diaphragm structure, and reduced adhesion between the basement membrane and the podocyte [9]. With the increasing understanding of ROS, therapeutic approaches targeting ROS are being tested in clinical trials [10]. Therefore, one possible promising therapeutic approach for NS is the removal of excessive ROS by new antioxidant nanomedicines.

As a new type of artificial enzyme, nanozymes feature unique advantages such as high stability, high catalytic activity, low cost and other unique properties of nanomaterials. Due to their wide application prospects, antioxidant nanozymes have been developed for various oxidative stress-related diseases [11]. Patients achieving remission is a key issue to clinical applications with a lower dosage as it helps to reduce adverse events. It is well known that ceria nanoparticles have been extensively used in oxidative stress-related diseases because of their catalytic activity and free radical scavenging as antioxidants [12–14]. Cerium dioxide nanoparticles are present in both Ce^3+^ to Ce^4+^ ions. Ce^3+^ removes hydroxyl (·OH) through redox reactions, removes superoxide anion (·O_2_^–^) by stimulating the activity of superoxide dismutase (SOD), while Ce^4+^ removes H_2_O_2_ by stimulating the activity of catalase (CAT) [15]. It has been displayed that Ce^3+^ ions are more important in scavenging ROS because they provide better removal of ·OH and ·O_2_^–^ which are related to cell death and inflammatory response [16, 17]. The ratio of Ce^3+^ to Ce^4+^ can be adjusted by the doping ions to improve the performance of ROS scavenging [18–20]. Zr^4+^ not only regulates the ratio of Ce^3+^ and Ce^4+^ but also controls the conversion rate between the two oxidation states, which can effectively scavenge ROS at a very low dose [18, 21].

**Scheme 1:**
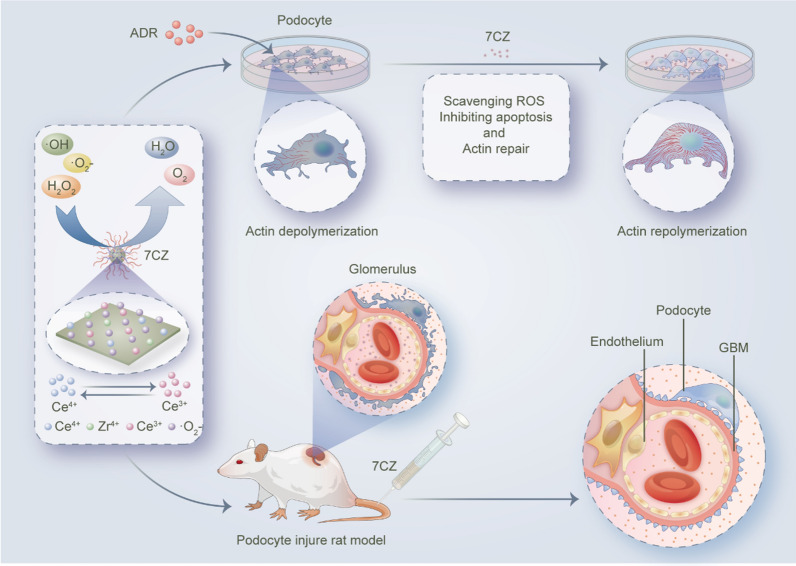
Schematic illustration of 7CZ nanomedicines in treating podocyte injury in vitro and in vivo

In this report, we rationally designed and engineered ceria-zirconia nanomedicines (CZ; Ce_x_Zr_1−x_O_2_, where x = 0.3, 0.5, 0.7, 0.9; named as 3CZ, 5CZ, 7CZ and 9CZ nanomedicines, respectively) as an enzyme-mimicking nano agent to explore the effect of the valence state of cerium in the matrix on its multiple antioxidant activities of nanozymes. It has been found that Ce_0.7_Zr_0.3_O_2_ (7CZ) nanomedicines feature significant ROS scavenging capability at a very low concentration. Importantly, the very low dosage of 7CZ nanomedicines was highly effective in protecting against oxidative stress-induced podocyte injury both in vivo and in vitro (Scheme 1). Thus, the effective therapeutic of 7CZ nanomedicines on podocyte injure rat models may shed new lights on the management of PNS by using nanotechnology.

## Results and discussion

### Synthesis and characteristics of CZ nanomedicines

**Fig. 1:**
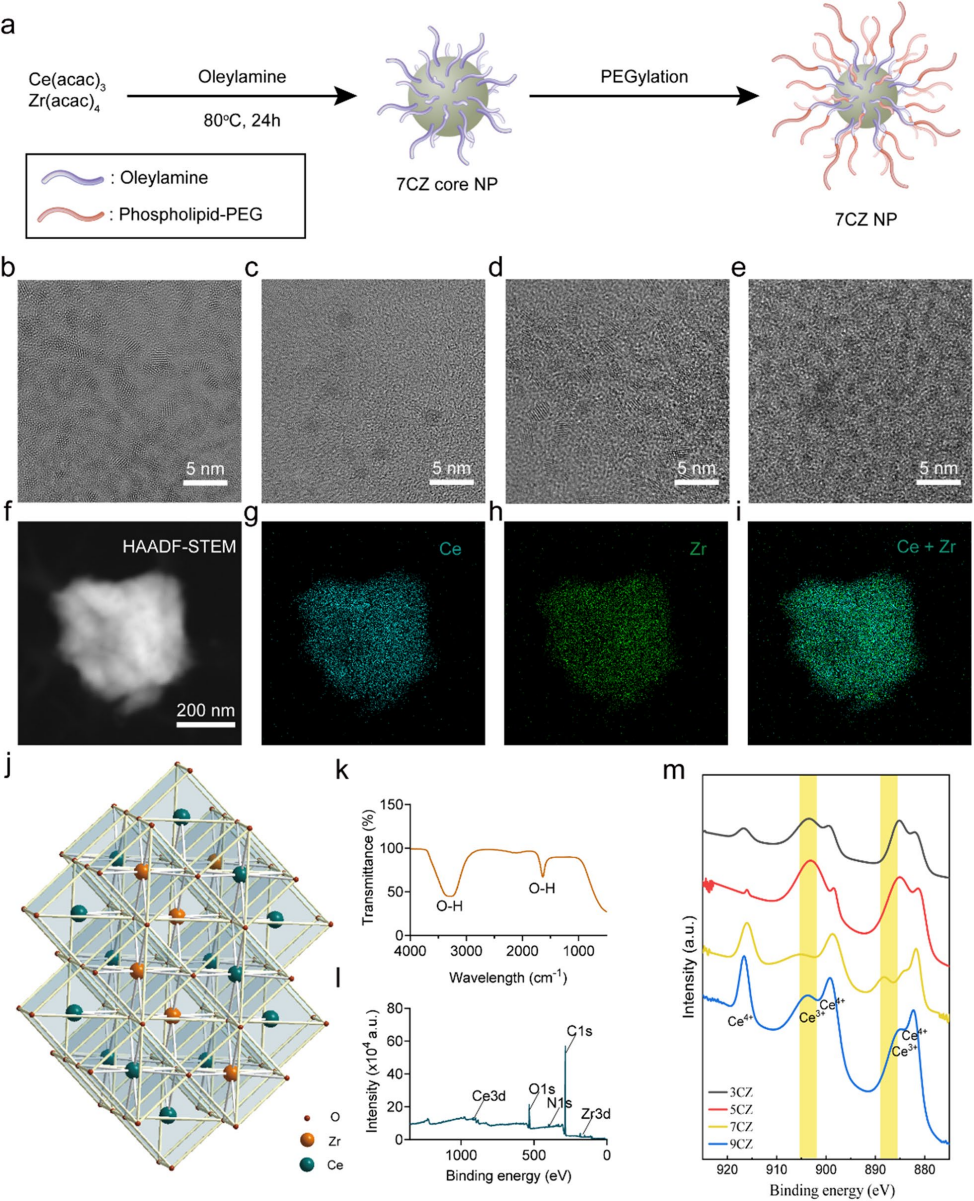
Synthesis and characterization of the CZ nanomedicines. **a** Schematic illustration of CZ nanomedicines. **b**–**e** TEM images of 3CZ (**b**), 5CZ (**c**), 7CZ (**d**), and 9CZ (**e**) nanomedicines, respectively. **f**–**i** EELS elemental mapping of Ce (blue) and Zr (green). **j** Schematic diagram of crystal structure models of 7CZ nanomedicines. The red, yellow and green balls represent O, Zr, and Ce atoms, respectively. **k** FTIR spectrum of 7CZ nanomedicines. **l** XPS spectrum of 7CZ nanomedicines. **m** XPS spectrum of the Ce in 3CZ, 5CZ, 7CZ and 9CZ nanomedicines. Ce^3+^ peaks are marked with yellow bands

A series of Zr^4+^-doped samples Ce_x_Zr_1−x_O_2_ (x = 0.3, 0.5, 0.7, 0.9) were fabricated by a non-hydrolytic sol-gel process with CeO_2_ as the precursors (Fig. 1a). The obtained nanoparticles were named as 3CZ, 5CZ, 7CZ, 9CZ nanomedicines, respectively. Transmission electron microscopy (TEM) images revealed that these samples (3CZ, 5CZ, 7CZ, and 9CZ nanomedicines) are similarly sized spherical nanoparticles, which excludes additional effects of specific topography on enzyme-like activity (Fig. 1b–e and Additional file 1: Fig. S1). Generalized high-angle annular dark field scanning transmission electron microscopy (STEM) (Fig. 1f–i) and energy-dispersive spectroscopy (EDS) (Additional file 1: Fig. S2) demonstrate the successful solid-solution formation in 7CZ nanomedicines. Typical ball-and-stick and polyhedral models are displayed in 7CZ nanocrystals (Fig. 1j). Dynamic light scattering (DLS) indicates that the average hydrodynamic diameter of the 7CZ nanomedicines is around 9 nm (Additional file 1: Fig. S3). The Zeta potential of 7CZ nanomedicines is − 6.48 mV in aqueous solution. The Fourier transform infrared (FTIR) spectrum of 7CZ nanomedicines revealed a characteristic O–H bending and stretching vibration at 3278 cm^−1^ and a peak at 1634 cm^−1^ corresponding to O-H of PEG (Fig. 1k). The X-ray photoelectron spectroscopy (XPS) analysis suggested that the successively synthesized 7CZ nanomedicines contain cerium and zirconium components (Fig. 1l). In addition, the peaks of cerium and zirconium components were detected, where the Ce^3+^-to-Ce^4+^ ratio of 7CZ nanomedicines is consistent with previous study (Fig. 1m and Additional file 1: Fig. S4) [22]. The satisfactory synthesis of 7CZ nanomedicines was further demonstrated by characterizing the crystalline phase of 7CZ nanomedicines using X-ray diffraction (XRD). As illustrated in Aditional file 1: Fig. S5, the diffraction peak of Ce at 44.51° can be attributed to (111) according to CeO_2_ (PDF#43-1002). The other diffraction peaks are not reflected due to the low crystallinity of the 7CZ nanomedicines. These results suggested that CZ nanomedicines with small sizes are successfully fabricated for further achieving the requirement of enhanced ROS scavenging.

### Multi-enzyme activity evaluation of CZ nanomedicines

**Fig. 2:**
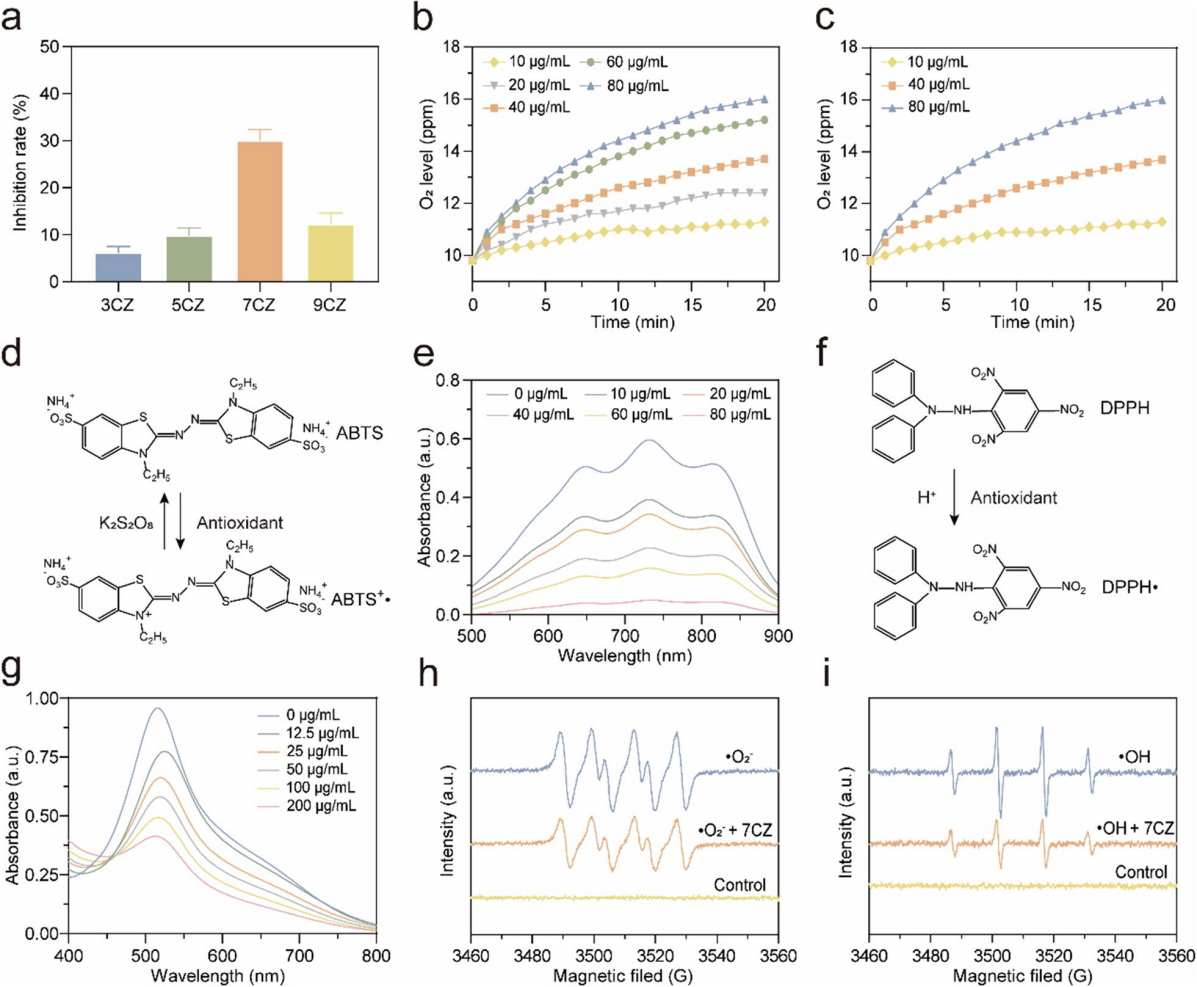
The multienzyme-mimetic abilities of CZ nanomedicines. **a** SOD-like activity of CZ nanomedicines. Effects of different concentrations of 7CZ nanomedicines (**b**) and H_2_O_2_ (**c**) on O_2_ production for CAT-like activity evaluation. **d**–**g** Two typical free radicals were monitored to measure the scavenging activity of 7CZ nanomedicines by UV–vis spectroscopy. **d**, **e** ABTS, **f**, **g** DPPH. ESR results of 7CZ nanomedicines for **h** ·O_2_^–^ and **i** ·OH elimination

To evaluate the antioxidant capacity of CZ nanomedicines, we tested different types of enzyme-mimicking activities. Based on the fact that SOD can catalyze ·O_2_^–^ to produce oxygen (O_2_) and hydrogen peroxide (H_2_O_2_) [23], the SOD enzyme activity of CZ nanomedicines was measured using the typical WST-8 method. The results shown that 7CZ nanomedicines feature a high ·O_2_^–^ scavenging ability (Fig. 2a) and the ·O_2_^–^ scavenging ability is enhanced with increased concentration (Additional file 1: Fig. S6). CAT is known to catalyze H_2_O_2_ to generate H_2_O and O_2_, therefore the O_2_ production was monitored with a dissolved oxygen probe to verify the CAT-mimicking ability of 7CZ nanomedicines [24]. The results displayed that O_2_ levels gradually increase with elevating concentrations of 7CZ nanomedicines (Fig. 2b) and H_2_O_2_ (Fig. 2c). 2,2′-Azino-bis (3-ethylbenzothiazoline-6-sulfonic acid) radical ion (ABTS^**.**+^) assay (Fig. 2d, e) and 2-2 diphenyl-1-picrylhydrazyl radical (DPPH·) assay (Fig. 2f, g) were used to estimate the overall antioxidant ability of 7CZ nanomedicines. Moreover, the free radical-scavenging capability is positively associated with the concentration of 7CZ nanomedicines (Additional file 1: Figs. S7, S8). These results indicated that 7CZ nanomedicines have excellent free radical-scavenging performance. In addition, we observed the capability of 7CZ nanomedicines on ·O_2_^–^ and ·OH scavenging by electron spin resonance (ESR) (Fig. 2h, i). With the addition of 7CZ nanomedicines, the ESR signal of the spin adducts decreased significantly. Notably, the 7CZ nanomedicines reflected high scavenging efficiency for both ·O_2_^–^ and ·OH.

### Protective effects of 7CZ nanomedicines in podocytes

**Fig. 3:**
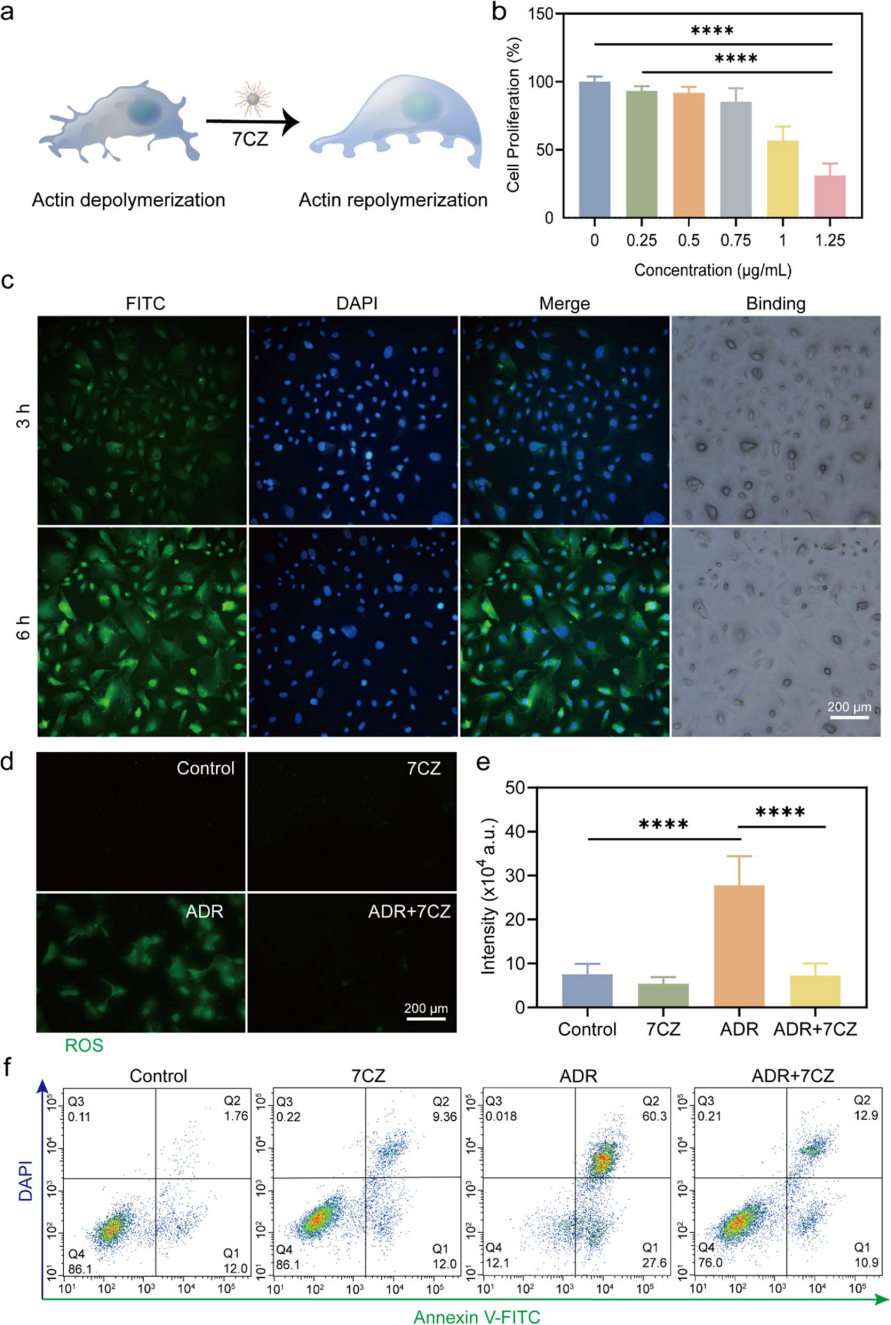
ROS scavenging activity of 7CZ nanomedicines in vitro. **a** Schematic illustration of the protective effect of 7CZ nanomedicines by redox-mediated ROS elimination. **b** Cytotoxicity of 7CZ nanomedicines in podocytes within 24 h. **c** Representative fluorescent microscope images of podocytes incubated with FITC-7CZ nanomedicines for 3 and 6 h are displayed. DAPI, blue; FITC-7CZ nanomedicines, green. Scale bar: 200 μm. **d** Intracellular ROS scavenging capability of 7CZ nanomedicines after different treatments for 24 h by DCFH-DA. Scale bar: 200 μm. **e** Qualitative analysis of ROS levels in podocytes after different treatments for 24 h. **f** Flow-cytometry-based apoptosis assay of cell apoptosis distribution in podocytes after different treatments. Data are presented as mean ± SD, one-way ANOVA, ns, non-significance, *P < 0.05, **P < 0.01; ***P < 0.001, ****P < 0.0001, vs. ADR group

In consideration of its remarkable multiple antioxidant activity and high biocompatibility of CZ nanomedicines, we further demonstrated the excellent protective effects of the optimized nanozyme (i.e., 7CZ) against redox-mediated elimination of ROS in adriamycin (ADR)-induced podocyte injury (Fig. 3a). The cytotoxicity of 7CZ nanomedicines was assessed using the Cell Counting Kit-8 (CCK-8) method. The results suggested that 7CZ nanomedicines induced significant cytotoxicity only when the concentration exceeded 0.75 µg/mL, which indicated the excellent biocompatibility of 7CZ nanomedicines (Fig. 3b). To test the uptake of 7CZ nanomedicines in podocytes, fluorescein isothiocyanate (FITC)-coupled 7CZ nanomedicines were co-cultured with podocytes for 3 and 6 h. The results indicated that a large number of 7CZ nanomedicines were engulfed by podocytes at 6 h (Fig. 3c). Podocytes were treated with ADR to mimic excessive ROS production. Intracellular ROS levels were measured by using 2′,7′-dichlorofluorescein diacetate (DCFH-DA) as a fluorescent probe. Fluorescence microscopy confirmed that 7CZ nanomedicines featured desirable intracellular ROS scavenging ability (Fig. 3d). Further quantitative analysis revealed the intracellular ROS level and its protective effect on podocytes (Fig. 3e). It has been indicated that podocyte apoptosis is attributed to PNS. The protective effect of 7CZ nanomedicines on ADR-induced podocyte apoptosis was measured by flow cytometry [25]. As shown in Fig. 3f, the highest percentage of apoptosis was observed in the ADR group. Early and late podocyte apoptosis was significantly inhibited after 7CZ nanomedicines treatment. The results of these in vitro studies demonstrated the protective effect of 7CZ nanomedicines both ROS and apoptosis in podocytes.

**Fig. 4:**
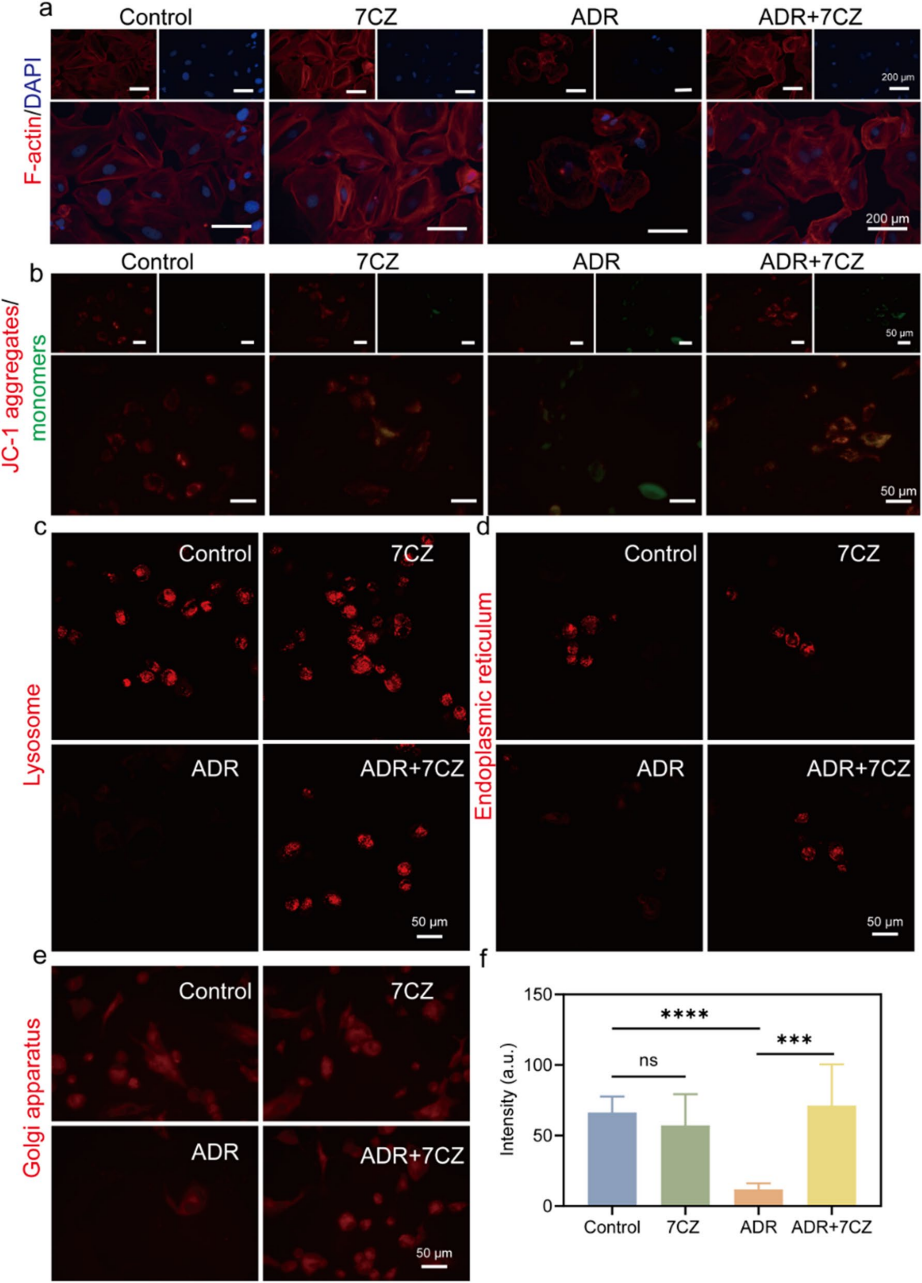
Protective effect of 7CZ nanomedicines on subcellular organelles. **a** Stress fibers of human kidney podocytes were observed by CLSM after different treatments. The stress fibers were stained red, the nuclei were stained blue. Changes of the mitochondrial membrane potential (**b**), lysosome (**c**), endoplasmic reticulum (**d**) and golgi apparatus (**e**) in podocytes after different treatments. **f** Quantitative analysis of the fluorescence intensities by Image J to evaluate the restoration of lysosome after injury. Data are presented as mean ± SD, one-way ANOVA, ns, non-significance, *P < 0.05, **P < 0.01; ***P < 0.001, ****P < 0.0001, vs. ADR group

Stabilizing the podocyte actin cytoskeleton is critical for the improvement of podocyte function [26]. The depolymerization of podocyte actin cytoskeleton leads to the loss of normal structure in foot processes under the condition of PNS, which is also known as podocyte foot effacement [27, 28]. We simulated PNS in ADR-induced podocyte injury in vitro. Actin cytoskeleton damage in podocytes was manifested by cell shrinkage and lack of actin stress fibers. This was consistent with the results in Fig. 4a. The results revealed that 7CZ nanomedicines significantly improved ADR-induced actin cytoskeleton damage. Mitochondria dysfunction is considered to be one of the pathogenes in renal diseases [29]. Mitochondria membrane potential (MMP) is a key indicator of mitochondria function, and a decrease in MMP is usually considered as a sign of early apoptosis [30]. The changes in MMP were observed using JC-1 staining, where the green fluorescence intensity was significantly increased in ADR-treated cells. Comparatively, it was largely decreased with the addition of 7CZ nanomedicines (Fig. 4b). It indicated the protective effects of 7CZ nanomedicines in mitochondrial function by scavenging ROS. The growing evidence has shown that normal lysosomal function plays an important role in keeping podocyte homeostasis [31]. To further validate the role of lysosomes in ADR-induced podocyte injury, lysosomal damage was evaluated using the Lysosome Tracker Red. We observed a significant enhancement of lysosomal markers with the addition of 7CZ nanomedicines, suggesting a strong protective effect of 7CZ nanomedicines on lysosomes (Fig. 4c, f). The endoplasmic reticulum (ER) is an interconnected network of membranes involved in protein synthesis, folding, and quality control. It is also responsible for lipid metabolism and calcium homeostasis [32]. The ER has a crucial role in maintaining redox balance through the expression of antioxidant proteins and enzymes. ER stress, often associated with an imbalance between ROS production and ROS scavenging systems, can trigger an unfolded protein response and lead to oxidative damage [33]. The Golgi apparatus has been implicated in the organization and trafficking of antioxidant enzymes that scavenge ROS [34]. The protective effect of 7CZ nanomedicines on the ER and Golgi apparatus was verified (Fig. 4d, e). These results solidly demonstrated that 7CZ nanomedicines could protect actin cytoskeleton and cellular organelles to maintain normal podocyte morphology and function.

### The underlying mechanism of 7CZ nanomedicines for the treatment of ADR-induced podocyte injury

**Fig. 5:**
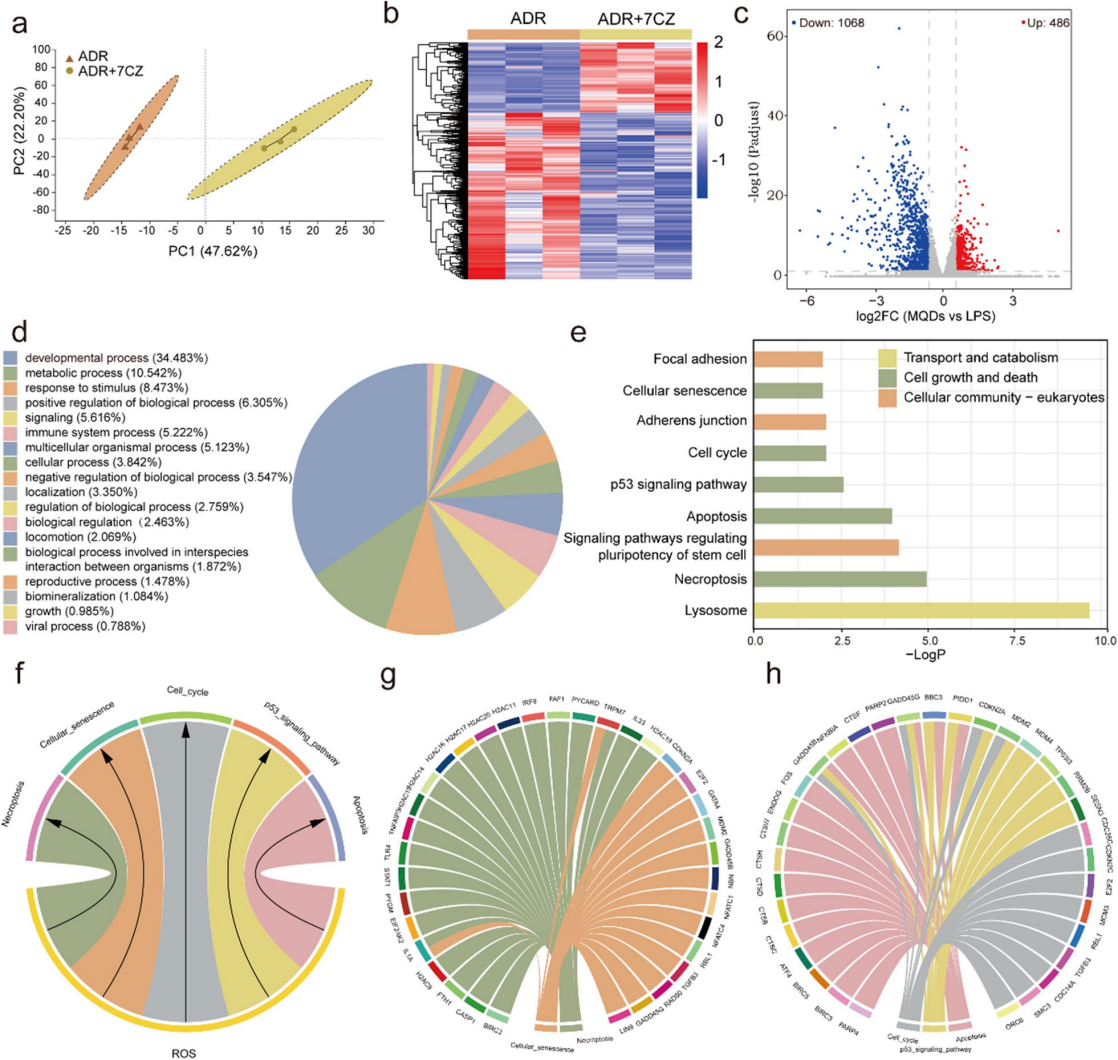
RNA sequencing of 7CZ nanomedicines application in ADR-treated podocytes. **a** PCA was performed based on differentially expressed genes with or without 7CZ nanomedicines. Each data point corresponds to the PCA analysis of each sample. **b** Heatmap of significant genes involved after 7CZ nanomedicines treatment (fold change ≥ 2 and *P* < 0.05). **c** Volcano plots showing the identified upregulated and downregulated genes by 7CZ nanomedicines. **d** GO analysis of the DEGs in NS after 7CZ nanomedicines treatment. **e** KEGG pathway enrichment analysis of the identified differentially expressed genes. **f** KEGG pathway enrichment analysis of differentially expressed genes associated with ROS. The 5 most significantly enriched pathways are shown in **g** and **h**

The mRNA sequencing was performed to elucidate the possible protective molecular mechanisms of 7CZ nanomedicines in ADR-induced podocyte injury. The no-guided principal component analysis (PCA) of the data displayed that there were differences between the groups (Additional file 1: Fig. S9), particularly in transcriptome profiles between ADR and ADR + 7CZ nanomedicines groups (Fig. 5a). Up-regulated and down-regulated genes were sifted and separated clearly by cluster analysis between Control and ADR groups (Additional file 1: Fig. S10). Meanwhile, 1554 genes were found to be differentially expressed between ADR and ADR + 7CZ nanomedicines groups, of which 486 and 1086 genes were up-regulated and down-regulated, respectively (Fig. 5b, c). According to Gene Ontology (GO) annotations, differentially expressed genes (DEGs) are annotated into the developmental process (34.483%), metabolic process (10.542%), response to stimulus (8.473%), positive regulation of biological process (6.305%), signaling (5.616%), etc. (Fig. 5d). Kyoto Encyclopedia of Genes and Genomes (KEGG) pathway enrichment analysis (Fig. 5e) displayed that the lysosome, necroptosis, signaling pathways regulating pluripotency of stem cell, apoptosis and p53 signaling pathway were closely associated with the therapeutic mechanisms of 7CZ nanomedicines. It also further elucidated the mechanism of ROS scavenging by 7CZ nanomedicines for NS treatment. In addition, the top 5 KEGG pathways associated with ROS enrichment were also listed based on the number of DEGs involved (Fig. 5f). Meanwhile, the top 5 ROS-related pathways and DEGs were listed separately in Fig. 5g, h according to KEGG analysis, including necroptosis, cellular senescence, cell cycle, p53 signaling pathway and apoptosis. The results demonstrated that these DEGs played an important role in the efficacy of 7CZ nanomedicines.

### Biocompatibility of 7CZ nanomedicines in vivo

**Fig. 6:**
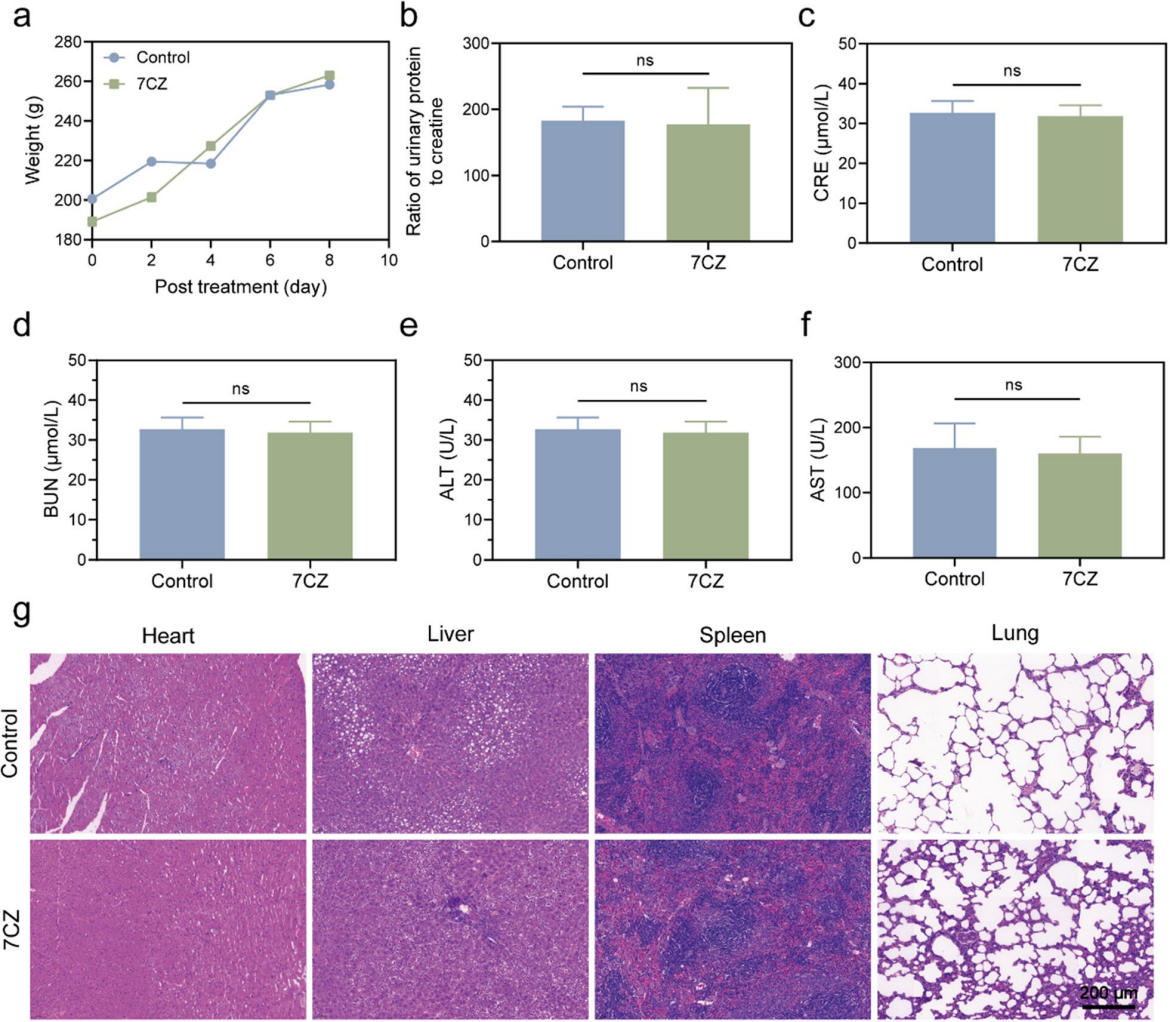
Biocompatibility of 7CZ nanomedicines in rats with ADR-induced nephrotic syndrome. **a** The levels of body weight were recorded in 8 days of post treatment (n = 5). The ratio of urinary protein to creatinine (**b**), serum CRE (**c**), BUN (**d**), ALT (**e**) and AST (**f**) after 7CZ nanomedicines i.p. injection (n = 5). **g** H&E staining of rat heart, liver, spleen, and lung, after treatment with 7CZ nanomedicines or normal saline for 8 days. The 7CZ nanomedicines group represents the ADR-induced NS treated with 0.1 mg/kg 7CZ nanomedicines every other day. Data are presented as mean ± SD, one-way ANOVA, ns, non-significance, *P < 0.05, **P < 0.01; ***P < 0.001, ****P < 0.0001, not significant, vs. ADR group

Before evaluating the effect of ROS scavenging in vivo, the biocompatibility of 7CZ nanomedicines was measured in rats. As shown in Fig. 6a, there was no notable weight loss after 7CZ nanomedicines treatment. The values of the ratio of urinary protein to creatinine (Fig. 6b) and all biochemical parameters including serum creatinine (CRE) (Fig. 6c), blood urea nitrogen (BUN) (Fig. 6d), alanine aminotransferase (ALT) (Fig. 6e) and aspartate aminotransferase (AST) (Fig. 6f) were within normal range after 7CZ nanomedicines treatment. In addition, hematoxylin and eosin (H&E) stained images of major organs such as the heart, liver, spleen and lung were used to evaluate the safety of 7CZ nanomedicines treatment. There were no significant morphological changes in rats after intravenous administration of 7CZ nanomedicines (Fig. 6g). These results confirmed the absence of systemic toxicity of 7CZ nanomedicines at the administered dose.

### Protective effects of 7CZ nanomedicines in rats with ADR-induced NS

**Fig. 7:**
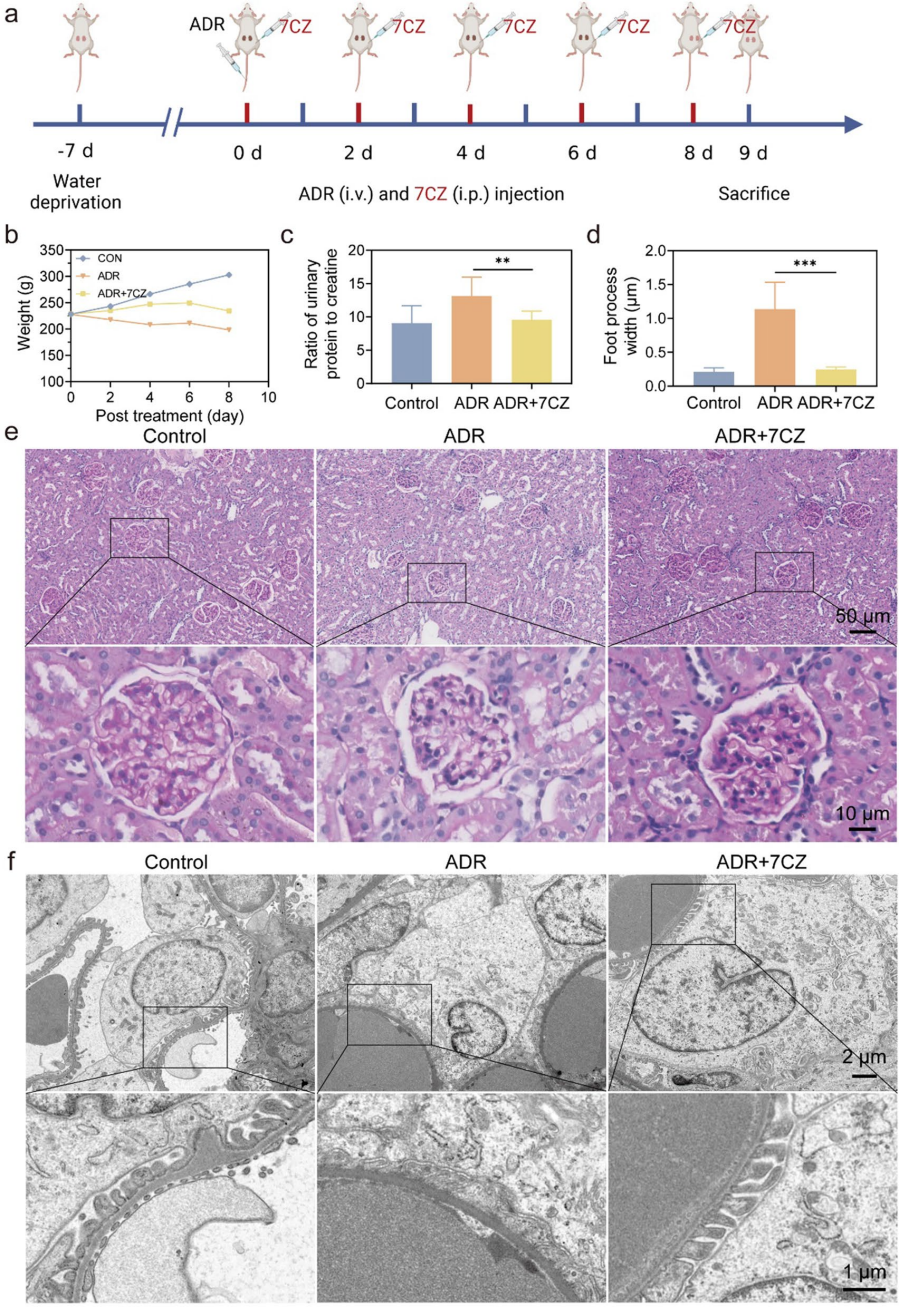
The efficacy of 7CZ nanomedicines in rats with ADR-induced nephrotic syndrome. **a** Schematic illustration for establishing nephrotic syndrome model and their treatment with 7CZ nanomedicines. **b** Changes in body weight were recorded in 8 days of post treatment. **c** The ratio of urine protein to creatinine after 8-day treatment. **d** Quantitative analysis of the foot process width by Image J. **e** PAS staining of the kidney after various treatments. **f** TEM of the morphology in podocytes was observed after different treatments. Data are presented as mean ± SD, one-way ANOVA, ns, non-significance, *P < 0.05, **P < 0.01; ***P < 0.001, ****P < 0.0001, vs. ADR group

The pathogenesis of the ADR-induced NS rat model is unclear. ROS plays an important role in podocyte injury in NS. Oxidative stress occurs when there is excessive production of ROS or insufficient antioxidant defense mechanisms. Excessive ROS can directly damage podocytes, triggering an inflammatory response and fibrosis, ultimately leading to impaired kidney function [8]. Based on the efficient scavenging of ROS in podocytes by 7CZ nanomedicines, their potential to treat oxidative stress-type diseases was explored in a rat model of NS. We evaluated the damage of ROS in ADR-induced NS rats by measuring body weight, renal function and histopathological changes. As presented in Fig. 7a, the NS rat model was established in rats with 7.5 mg/kg of ADR by tail vein injection after adaptive feed. Then, each rat was intraperitoneally injected with 0.1 mg/kg of 7CZ nanomedicines for 5 alternate days (Day 0, 2, 4, 6 and 8). The 7CZ nanomedicines treatment significantly increased the body weight of rats which was decreased by ADR (Fig. 7b). The ratio of urine protein to creatinine is a key indicator of podocyte injury in vivo. The results displayed that the ratio of urine protein to creatinine was significantly reduced after 7CZ nanomedicines treatment, suggesting that 7CZ nanomedicines can effectively improve damaged renal function in NS rats (Fig. 7c). In addition, there were no significant pathological changes in glomerulus in Periodic Acid-Schiff (PAS) staining (Fig. 7e). Actin skeleton disruption in podocytes leads to fusion of peduncles. The capability of 7CZ nanomedicines for preventing podocyte injury from ROS damage was further verified by TEM. The TEM images revealed podocyte foot process effacement and thickening of the basement membrane in ADR-induced NS rats. It was recovered after 8 days of 7CZ nanomedicines treatment (Fig. 7f). The foot process width was normalized after 7CZ nanomedicines treatment (Fig. 7d). It suggested that the alteration of podocyte function and morphology contributed to the development of proteinuria in PNS. These results displayed that 7CZ nanomedicines could efficiently alleviate podocyte injury in rats with ADR-induced NS.

## Conclusions

In summary, we engineered ultra-small 7CZ nanomedicines to protect against podocyte injury. By doping Zr^4+^ ions, the ratio of Ce^3+^ to Ce^4+^ and the conversion rate between the two oxidation states were controlled to improve their antioxidant properties thereby successfully achieving an effective reduction of oxidative stress. SOD/CAT-like properties of 7CZ nanomedicines can produce O_2_ by scavenging ·O_2_^–^ and H_2_O_2_ thereby alleviating the damage of excessive ROS in podocytes [35]. We demonstrated that just a low dosage of 7CZ nanomedicines is sufficient to protect podocytes from ROS damage in vitro and in vivo NS models. It is effective in improving renal function and alleviating podocyte injury with a low dosage of 7CZ nanomedicines in rats with ADR-induced NS. This study not only shed new lights on the development of highly efficient nanoenzymes with multiple antioxidant activities but also the management of PNS by using nanotechnology.

## Materials and methods

### Materials

Cerium(III) acetylacetonate hydrate and zirconium(IV) acetylacetonate (97%) were purchased from Sigma Aldrich (St. Louis, MO). Oleylamine (approximate C18 content of 80–90%) was purchased from Aladdin (Shanghai, China). Acetone (99.5%, extra pure), chloroform and H_2_O_2_ (30%) (99.5%, extra pure) were obtained from Shanghai University. FITC was supplied by Shanghai Runcheng Bio-tech Co. 1,2-Distearoyl-*sn*-glycero-3-phosphoethanolamine-*N*-[methoxy (polyethylene glycol)-2000] (mPEG (2000)-PE), 1,2-distearoyl-*sn*-glycero-3-phosphoethanolamine-*N*-[amino (polyethylene glycol)-2000] (DSPE-PEG (2000)-amine) and cyanine5.5 (Cy5.5) NHS ester were purchased from Rui Xi Biology (Xi-an, China). SOD assay kit, CCK-8, 4,6-diamidino-2-phenylindole (DAPI), DCFH-DA, JC-1, Lyso-tracker Red, ER-Tracker Red and Golgi-Tracker Red were purchased from Beyotime Institute of Biotechnology (Shanghai, China). Annexin V-FITC/PI Apoptosis Detection Kits were purchased from Yeasen (Shanghai, China).

### Synthesis of 2 nm ceria-zirconia nanomedicines

A 0.5 g mixture of hydrated cerium acetylacetonate and zirconium acetylacetonate in an appropriate molar ratio was added to 15 mL of oleylamine. The mixture was sonicated at 20 °C for 15 min and then heated to 80 °C at a rate of 2 °C/min. The reaction mixture was aged at 80 °C for 24 h and then cooled to room temperature. The mixture was washed with acetone (100 mL) at 5000 rpm/min several times and the obtained CZ nanomedicines precipitate was dispersed in chloroform at a final concentration of 10 mg/mL.

### Synthesis of phospholipid-polyethylene glycol-encapsulated CZ nanomedicines

The CZ nanomedicines dispersed in chloroform were dissolved in water by wrapping them with phospholipid polyethylene glycol (PEG). First, 5 mL of chloroform CZ nanomedicines (10 mg/mL) and 10 mL of chloroform mPEG (2000)-PE (10 mg/mL) were mixed. The mixture was spin evaporated in a rotary evaporator and then incubated in a vacuum oven at 70 °C for 2 h to remove the chloroform completely. Then, 5 mL of deionized water was added to the sample, which was filtered through a 0.4 μm filter and ultracentrifuged to remove excess mPEG (2000)-PE. Finally, the purified phospholipid-PEG-capped samples were dispersed in DI water.

### FITC-, or Cy5.5-NHS ester-conjugated of CZ nanomedicines

The nanomedicines were encapsulated on an amine-functionalized phospholipid-peg shell layer to attach FITC-, or Cy5.5-NHS ester to its surface. First, 5 mL of chloroform (10 mg/mL) CZ nanomedicines, 9 mL of chloroform mPEG (2000)-PE (10 mg/mL) and 1 mL of chloroform DSPE-PEG (2000) amine (10 mg/mL) were mixed. A purification step using phospholipid-peg encapsulated CZ nanomedicines was used to obtain CZ nanomedicines dispersed in DI water. Then, 5 mg of the selected dye was added to the CZ nanomedicines suspension and stirred at 40 °C for 12 h. After filtration and ultracentrifugation, the FITC- or Cy5.5-NHS ester-coupled CZ nanomedicines dispersed in DI water were obtained.

### Characterizations of phospholipid-PEG-capped CZ nanomedicines

The dispersed 7CZ nanomedicines dispersion was dropped onto the copper grid covered with carbon film, dried at room temperature and analyzed by TEM and STEM at 200 kV (JEM-2100f, JEOL, Japan). EDS analysis was performed with a single drift detector (X-MaxN, Oxford Instruments, UK). The hydrodynamic diameter of the samples was measured by DLS using a Zetasizer Nano-ZS system (Malvern Instruments Ltd., Malvern, UK). Zeta potential was measured in water through a Mastersizer 3000 (Malvern, Britain) laser diffraction particle size analyzer. FTIR was performed using a FTIR-8300 series spectrometer for spectral analysis (Shimadzu, Japan). The XPS was performed by an ESCALAB 250 Xi Mg (Thermo Scientific, Japan) X-ray source. The XRD measurements were obtained with a diffractometer (New D8 Advance, Bruker, Germany).

### SOD mimetic activity assay

The SOD-like activity of CZ NPs was measured using a total SOD activity assay kit with the WST-8 method (Beyotime, China) according to the manufacturer’s instructions. The absorbance was estimated at 450 nm using a microplate reader.

### CAT mimetic activity assay

The CAT-like activity of CZ nanomedicines was assessed by measuring O_2_production using a JPB-607 A portable dissolved oxygen meter (INESA Scientific Instruments, Shanghai, China). Briefly, 2 mL solutions of 7CZ nanomedicines at different concentrations were added to 8 mL of PBS containing H_2_O_2_ or 2 mL solutions of 7CZ nanomedicines mixed with 8 mL of PBS containing H_2_O_2_ at different concentrations. The reaction was performed at room temperature for 20 min, and the production of O_2_ was measured at the interval of 1 min.

### Evaluation of the antioxidation and reproducibility were detected by ESR

All ESR experiments were performed using a Bruker EMX ESR spectrometer (Billerica, MA) at room temperature. ·OH were generated in the Fenton reaction (20 µL of 0.735 mM FeSO_4_ and 30 µL of 0.315 mM H_2_O_2_). ·O_2_^–^ were generated by irradiation with riboflavin solution. The efficiency of 7CZ nanomedicines on the removal of ·OH/·O_2_^–^ was evaluated by ESR spectroscopy (JEOL-FA200) when DMPO was used as the spin trapping agent (4 µL, 98%). The scavenging ability of 7CZ nanomedicines for ROS is represented by the change in relative peak intensity of the ESR spectrum.

### Scavenging ABTS radicals

The ability of 7CZ nanomedicines to scavenge ABTS radical cation (·ABTS^+^) radicals was measured by using the ABTS radical cation decolorization assay. 7.4 mmol ABTS was reacted with 2.6 mM potassium persulfate in deionized water to generate ·ABTS^+^ and kept in a dark place for 24 h at room temperature. UV–vis spectra were measured at 734 nm after ·ABTS^+^ reacted with 7CZ nanomedicines solutions of different concentrations for 5 min. The inhibition rate of ·ABTS^+^ free radicals was calculated according to the ratio of neutralized ·ABTS^+^ free radicals to total free radicals. All experiments were performed in triplicate.

### Scavenging DPPH radicals

The ability of 7CZ nanomedicines to scavenge DPPH radicals was measured by using the DPPH radical cation decolorization assay. 3 mmol of DPPH was dissolved in anhydrous ethanol. UV–vis spectra were measured at 517 nm after DPPH radical reacted with 7CZ nanomedicines of different concentrations for 30 min. The inhibition rate of DPPH free radicals was calculated according to the ratio of neutralized DPPH free radicals to total free radicals. All experiments were performed in triplicate.

### Cell culture

Conditionally immortalized human podocytes AB8/13 (University of Bristol, UK) were grown in RPMI-1640 medium supplemented with 10% fetal bovine serum and 1% penicillin/streptomycin. Human podocytes supplemented with insulin-transferrin-selenium (Life Technologies) proliferated at 33 °C. Fully differentiated human podocytes were used for experiments after culturing at 37 °C for 10–14 days.

### Cell viability assay

The viability of human kidney podocytes was determined using the CCK-8 assay. Podocytes differentiated at 37 °C for 13 days were inoculated in 96-well plates at 1.0 × 10^4^ cells per well and incubated for 24 h at 37 °C, 5% CO_2_. The prepared 7CZ nanomedicines suspension was dispersed in the cell culture medium at appropriate concentrations so that the final concentrations were 0, 0.25, 0.5, 0.75, 1.0, 1.25 µg/mL and incubated at 37 °C for 24 h. After 24 h, 100 µL of a mixture of CCK-8 and serum-free medium (1:9 volume of CCK-8 solution: serum-free medium) was added to each well. The incubation was continued for 1 h under the same conditions, and the absorbance was measured spectrophotometrically at OD = 460 nm.

### Fluorescence microscopy of cellular uptake

To evaluate the cellular uptake of nanoparticles, FITC-coupled 7CZ nanomedicines (12.5 µg/mL) were added to the podocyte culture medium (12-well plates at a density of 1.2 × 10^5^ cells/well; n = 4 per group). After incubation for 3 and 6 h at 37 °C with 5% CO_2_ in the dark, cells were washed three times with phosphate-buffered saline (PBS). After being fixed with 2.5% paraformaldehyde (PFA), cells were stained with 4,6-diamidino-2-phenylindole (DAPI) and observed under fluorescence microscopy.

### Measurement of ROS levels

Intracellular ROS levels were monitored by fluorescence microscopy using DCFH-DA as a fluorescent probe. Podocytes were inoculated on 12-well plates and incubated at 37 °C with 5% CO_2_ for 24 h at a density of 1.2 × 10^5^ per well. After washing with PBS, ADR or 7CZ nanomedicines were incubated with the podocytes for a further 24 h. DCFH-DA was diluted with serum-free medium to the appropriate concentration and co-incubated with the cells in the dark for 20 min. The cells were washed three times with cell culture medium and then observed under a fluorescence microscope. Fluorescence quantification was performed using Image J software.

### Apoptosis analysis

The proportion of apoptotic cells was measured with the Annexin V-FITC/DAPI Apoptosis Detection Kit (Yeasen, Shanghai, China). Podocytes were inoculated on 6-well plates and incubated at 37 °C with 5% CO_2_ for 24 h at a density of 2.4 × 10^5^ per well. Cells were treated with fresh medium (control), ADR (1 µg/mL), ADR (1 µg/mL) + 7CZ nanomedicines (0.75 µg/mL), and 7CZ nanomedicines (0.75 µg/mL) for 24 h at 37 °C with 5% CO_2_. Podocytes were collected and washed twice with PBS. Cells were resuspended with 1× Binding Buffer and mixed with 5 µL Annexin V-FITC and 10 µL DAPI. Finally, the reaction was carried out in the dark at room temperature for 15 min and detected by flow cytometry (Molecular Devices, America).

### Actin cytoskeleton of podocytes

To investigate the stability of ADR-induced podocyte actin cytoskeleton depolymerization after 7CZ nanomedicines treatment. Podocytes were inoculated on 12-well plates and incubated at 37 °C with 5% CO_2_ for 24 h at a density of 1.2 × 10^5^ per well. Cells were treated with fresh medium (Control), ADR (1 µg/mL), ADR (1 µg/mL) + 7CZ nanomedicines (0.75 µg/mL), and 7CZ nanomedicines (0.75 µg/mL) for 24 h at 37 °C with 5% CO_2_. Cells were fixed in 4% paraformaldehyde for 10 min and washed three times with PBS. After fixation, the cells were permeabilized with PBS containing 0.1% triton X-100 for 30 min at room temperature and washed three times again with PBS. The cells were blocked with PBS containing 1% BSA for 15 min and washed three more times. TRITC-phalloidin (100 nM, 300 µL) was added for 30 min at room temperature. DAPI was used as a stain for 10 min, and PBS was washed three times. Changes in the actin cytoskeleton of podocytes were observed under fluorescence microscopy.

### Subcellular staining

Podocytes were inoculated on 12-well plates and incubated at 37 °C with 5% CO_2_ for 24 h at a density of 1.2 × 10^5^ per well. Then staining with JC-1, Lyso-tracker Red, ER-Tracker Red and Golgi-Tracker Red. Podocytes were visualized using fluorescence microscopy and confocal laser scanning microscope. Fluorescence quantification was performed using Image J software.

### The RNA-sequencing assay

Library construction and sequencing were performed at Shanghai Majorbio Bio-pharm Technology Co., Ltd (Shanghai, China). The library was constructed using the Illumina TruseqTM RNA Sample Preparation Kit. A total of 1 mg RNA was extracted by using the TRIZOL reagent and then Illumina NovaSeq6000 sequencing was performed. RNA quality and quantification were measured using the 2100 Bioanalyzer (Agilent Technologies) and ND-2000 software (NanoDrop Technologies). Isolation of messenger RNA according to polyA selection and fragmentation by using fragmentation buffer. Then, double-stranded cDNA was synthesized by a SuperScript double-stranded cDNA synthesis kit (Invitrogen, CA) with random hexamer primers (Illumina). End-repair, phosphorylation and ‘A’ base addition according to the synthesized cDNA were performed according to Illumina’s library construction procedure. Following PCR amplification and TBS380 quantification using Phusion DNA polymerase (NEB), the libraries were subjected to paired-end RNA-seq sequencing using an Illumina Nova Seq 6000 sequencer (2 × 150 bp read length). Use fastp (https://github.com/OpenGene/fastp) to trim and quality control raw paired-end reads with default parameters [36]. Clean reads were individually compared to the reference genome in targeted mode using HISAT2 (https://ccb.jhu.edu/software/hisat2/index.shtml) [37] software. The mapped reads for each sample were constructed by String Tie (https://ccb.jhu.edu/software/stringtie/) on a reference basis [38]. DEGs between two different samples/groups were identified based on the Transcripts Per Million Reads (TPM) method for calculating the expression level of each gene. Gene abundance was quantified using RSEM (https://deweylab.biostat.wisc.edu/rsem/) [39]. Additionally, functional enrichment analyses involving GO (Gene Ontology, https://www.geneontology.org) and KEGG (Kyoto Encyclopedia of Genes and Genomes, https://www.genome.jp/kegg/) were performed to determine which DEGs were enriched significantly for GO terms and metabolic pathways at P-adjust ≤ 0.05 compared to a transcriptome-wide background. Goatools (https://github.com/tanghaibao/Goatools) and KOBAS (https://kobas.cbi.pku.edu.cn/home.do) were used for GO functional enrichment and KEGG pathway analysis [40]. All alternative clipping events occurring in the sample using the recently released rMATS program (https://rnaseq-mats.sourceforge.net/index.html) [41]. Only isozymes similar to the reference or containing novel splice junctions were taken into account, and splicing differences were examined as exon inclusion, exclusion, substitution 5′, 3′, and intron retention events.

### ADR-induced nephrotic syndrome rat model

SD rats (male, 6 weeks old, 200–250 g) were obtained from Shanghai Slack Experimental Animal Co. Rats were given water and feed at relative humidity (45–55%), room temperature (20–24 °C), and a dark light cycle for 12 h. All animal experiments were approved by the Animal Ethics Committee of Shanghai Children’s Hospital. NS was induced in rats with a single tail vein injection of ADR (7.5 mg/kg).

### Biocompatibility analysis of 7CZ nanomedicines

To assess the biocompatibility of 7CZ nanomedicines, we evaluated body weight, urinary protein and creatinine, liver and kidney functions, and H&E staining of the heart, liver, spleen and lung in each group. The body weight of ADR-induced NS rats was measured 8 days after 7CZ nanomedicines treatment. Rats were executed on Day 8, blood and urine samples were collected. Blood samples were collected into centrifuge tubes, placed at rest and then centrifuged at 4 °C for 15 min with 2000×*g* to obtain serum. Serums were used to evaluate liver and kidney function. The heart, liver, spleen and lungs were collected and fixed in paraformaldehyde (4% in PBS). These fixed samples were used for paraffin embedding, sectioning and H&E staining.

### Evaluation of the efficacy of 7CZ nanomedicines

To evaluate the efficacy of 7CZ nanomedicines in rats with ADR-induced NS, we assessed body weight, urinary protein and creatinine levels, PAS staining and ultrastructure of kidney tissues under TEM. The body weight of NS rats was monitored 8 days after 7CZ nanomedicine treatment. Rats were sacrificed on Day 9 and urine was collected. Urine was used for evaluating proteinuria. The kidney tissue fixed with paraformaldehyde was used for PAS staining. The kidney tissue fixed with 2.5% glutaraldehyde was used for PAS staining and TEM. Foot process width was measured using Image J software.

### Statistical analysis

All data are displayed as mean ± standard deviation (mean ± SD). Student’s t-tests were applied to analyze the statistical results (ns, non-significance; *P < 0.05; **P < 0.01; ***P < 0.001; ****P < 0.0001). *P* < 0.05 was considered to be statistically significant. All data were analyzed using GraphPad Prism (version 8.0, GraphPad Software, San Diego, CA) and R (version 3.5.1).

### Supplementary Information

**Additional file 1: Figure S1.** High (up column, scale bar = 20 nm) and low-magnification TEM images (down column, scale bar = 100 nm) of CZ nanomedicines. (a, e) 3CZ nanomedicines; (b, f) 5CZ nanomedicines; (c, g) 7CZ nanomedicines; (d, h) 9CZ nanomedicines. **Figure S2.** EDS spectra of the CZ nanomedicines. (a) 3CZ nanomedicines; (b) 5CZ nanomedicines; (c) 7CZ nanomedicines; (d) 9CZ nanomedicines. **Figure S3.** Hydrodynamic diameters of 7 CZ nanomedicines in water were measured by DLS. **Figure S4.** XPS spectra of the Zr 3d states in CZ nanomedicines. **Figure S5.** XRD spectra of the CZ nanomedicines. **Figure S6.** ·O_2_^−^ scavenging ability of 7CZ nanomedicines with different concentrations. **Figure S7.** Qualitative analysis of ABTS radicals scavenging activity of 7CZ nanomedicines with different concentrations. **Figure S8.** Qualitative analysis of DPPH radicals scavenging activity of 7CZ nanomedicines with different concentrations. **Figure S9.** The no-guided principal component analysis (PCA) of different groups. **Figure S10.** Heatmap of significant genes involved between control and ADR groups (fold change ≥ 2 and *P* < 0.05).

## Data Availability

The datasets and materials used in the study are available from the corresponding author.
