# Supplementary material for: Engineering antioxidant ceria-zirconia nanomedicines for alleviating podocyte injury in rats with adriamycin-induced nephrotic syndrome
Source: J Nanobiotechnology. 2023 Oct 19;21:384. doi: 10.1186/s12951-023-02136-2 (PMC10588015; doi:10.1186/s12951-023-02136-2)
Supplement: Supplementary file 1 — Additional file 1: Figure S1. High (up column, scale bar = 20 nm) and low-magnification TEM images (down column, scale bar = 100 nm) of CZ nanomedicines. (a, e) 3CZ nanomedicines; (b, f) 5CZ nanomedicines; (c, g) 7CZ nanomedicines; (d, h) 9CZ nanomedicines. Figure S2. EDS spectra of the CZ nanomedicines. (a) 3CZ nanomedicines; (b) 5CZ nanomedicines; (c) 7CZ nanomedicines; (d) 9CZ nanomedicines. Figure S3. Hydrodynamic diameters of 7 CZ nanomedicines in water were measured by DLS. Figure S4. XPS spectra of the Zr 3d states in CZ nanomedicines. Figure S5. XRD spectra of the CZ nanomedicines. Figure S6. ·O2− scavenging ability of 7CZ nanomedicines with different concentrations. Figure S7. Qualitative analysis of ABTS radicals scavenging activity of 7CZ nanomedicines with different concentrations. Figure S8. Qualitative analysis of DPPH radicals scavenging activity of 7CZ nanomedicines with different concentrations. Figure S9. The no-guided principal component analysis (PCA) of different groups. Figure S10. Heatmap of significant genes involved between control and ADR groups (fold change ≥ 2 and P < 0.05). [file 12951_2023_2136_MOESM1_ESM.docx]

**Additional file 1:**

**Engineering antioxidant ceria-zirconia nanomedicines for alleviating podocyte injury in rats with adriamycin-induced nephrotic syndrome**

Lili Liu^1,2^, Meiqi Chang^3^, Rong Yang^4^, Li Ding^5*^, Yu Chen^6*^, Yulin Kang^2*^

^1^School of Medicine, Anhui University of Science and Technology, Huainan, 232000, P. R. China.

^2^Department of Nephrology and Rheumatology, Shanghai Children’s Hospital, School of Medicine, Shanghai Jiao Tong University, Shanghai, 200062, P. R. China. E-mail: kangyl@shchildren.com.cn

^3^Laboratory Center, Shanghai Municipal Hospital of Traditional Chinese Medicine, Shanghai University of Traditional Chinese Medicine, Shanghai, 200071, P. R. China.

^4^Department of Pediatrics, Shanghai Tenth People’s Hospital, School of Medicine, Tongji University, Shanghai, 200072, P. R. China.

^5^Department of Medical Ultrasound, National Clinical Research Center of Interventional Medicine, Shanghai Tenth People's Hospital, Tongji University Cancer Center, Tongji University School of Medicine, Tongji University, Shanghai, 200072, P. R. China. E-mail: dingli@tongji.edu.cn

^6^ Materdicine Lab, School of Life Sciences, Shanghai University, Shanghai, 200444, P. R. China. E-mail: chenyuedu@shu.edu.cn

**Additional figures**

**
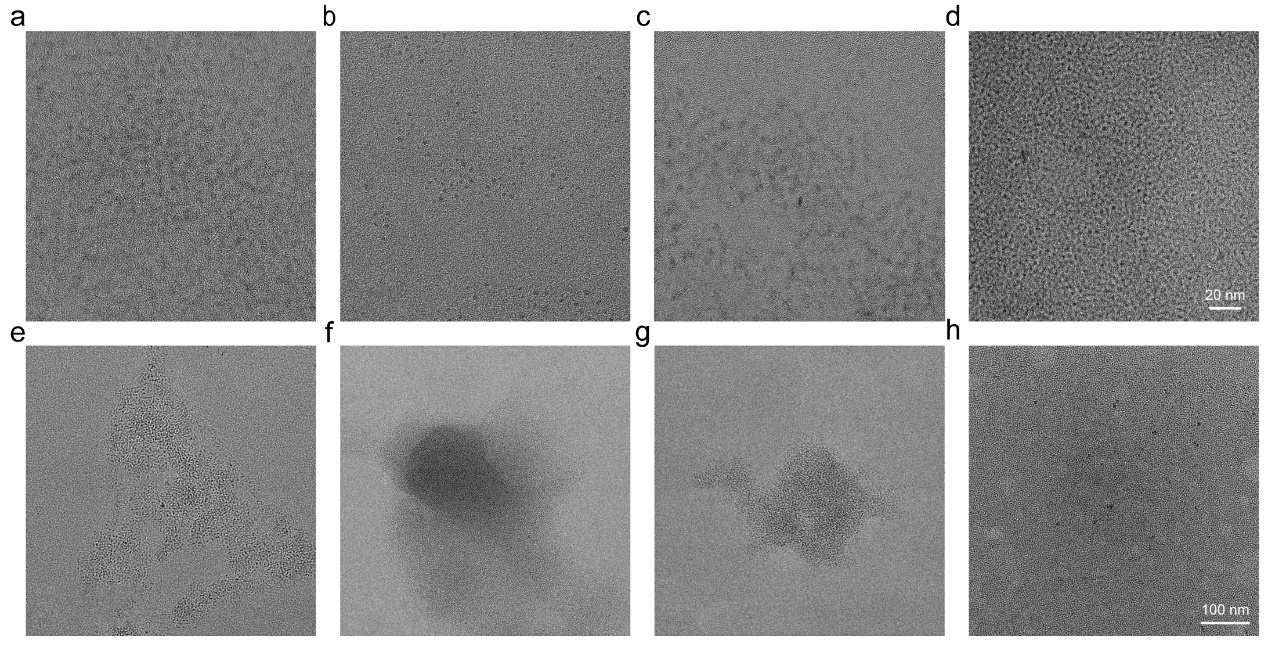
**

**Fig. S1** High (up column, scale bar =20 nm) and low-magnification TEM images (down column, scale bar = 100 nm) of CZ nanomedicines. (a, e) 3CZ nanomedicines; (b, f) 5CZ nanomedicines; (c, g) 7CZ nanomedicines; (d, h) 9CZ nanomedicines.

**
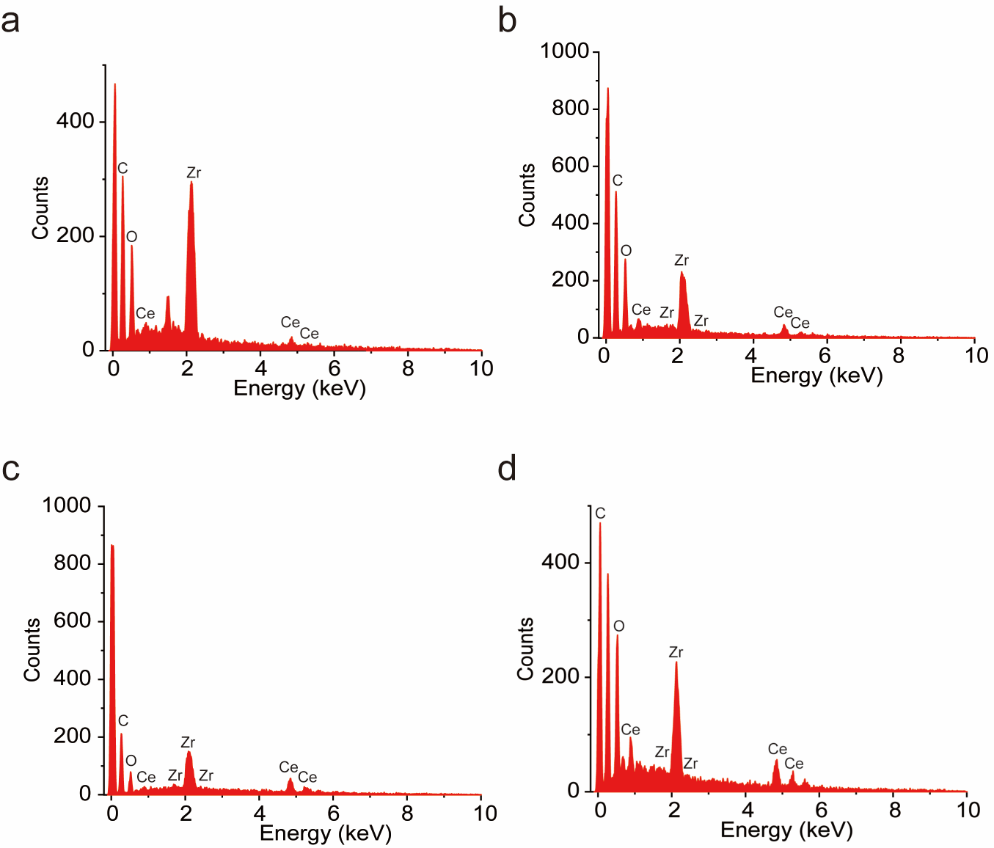
**

**Fig. S2** EDS spectra of the CZ nanomedicines. (a) 3CZ nanomedicines; (b) 5CZ nanomedicines; (c) 7CZ nanomedicines; (d) 9CZ nanomedicines.


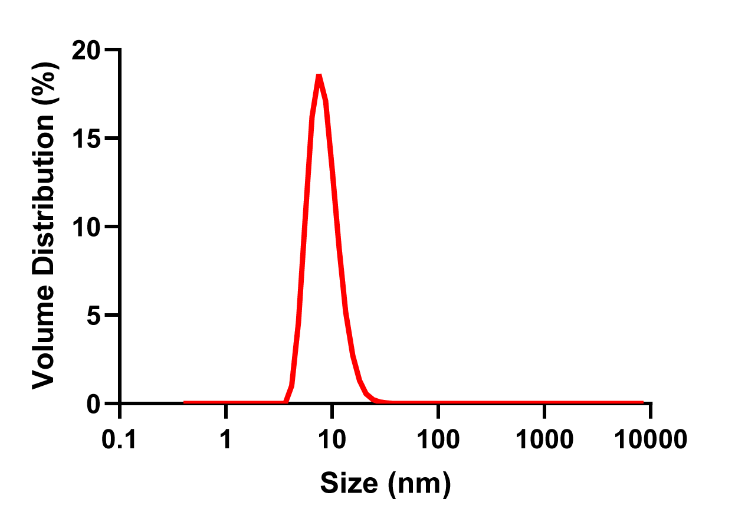


**Fig. S3** Hydrodynamic diameters of 7 CZ nanomedicines in water were measured by DLS.


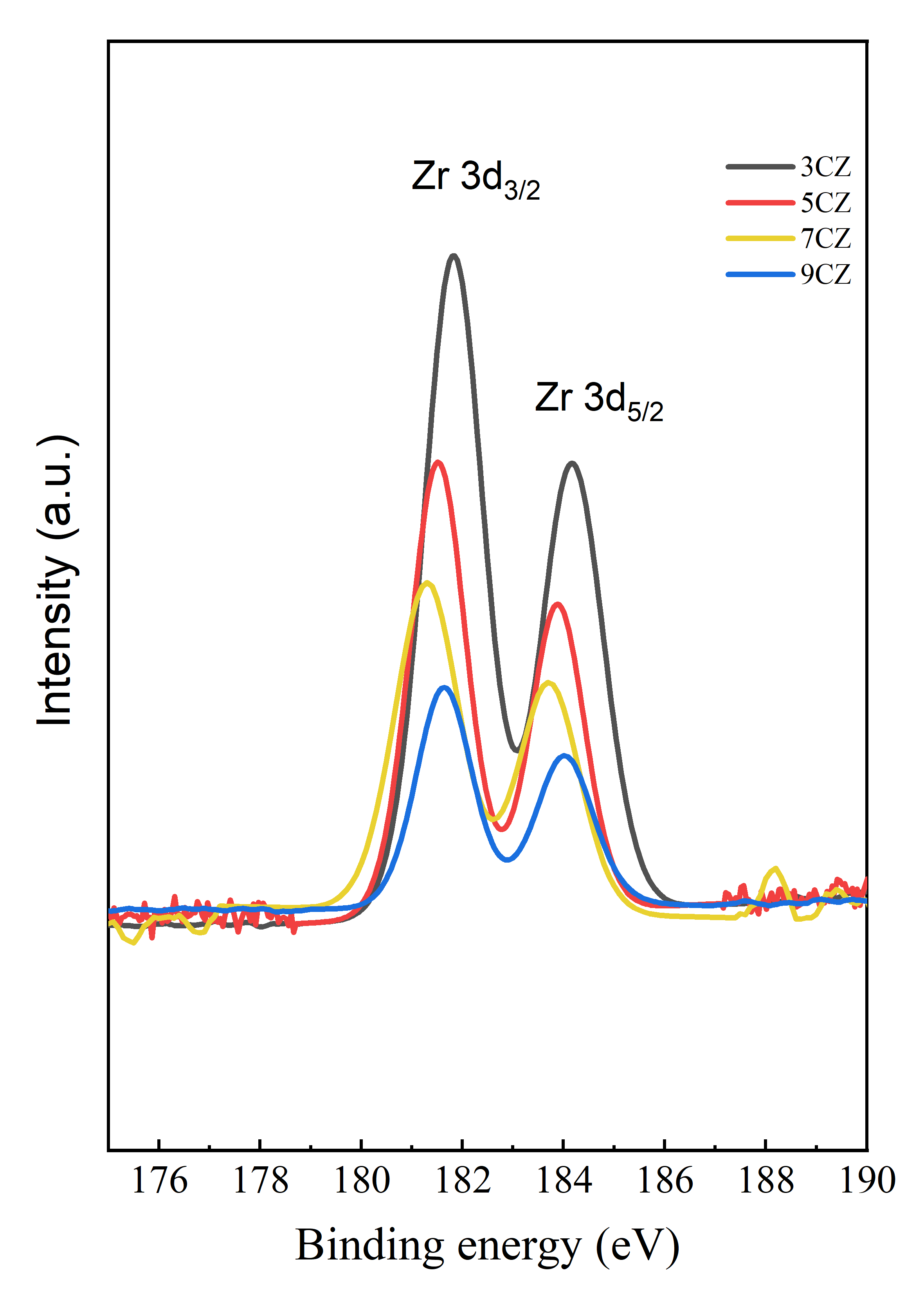


**Fig. S4** XPS spectra of the Zr 3d states in CZ nanomedicines.

**
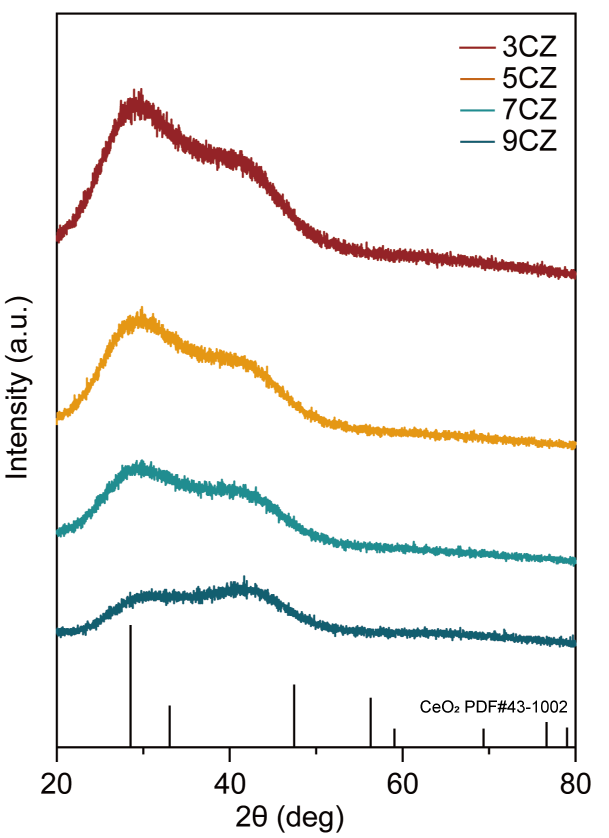
**

**Fig. S5** XRD patterns of the CZ nanomedicines.


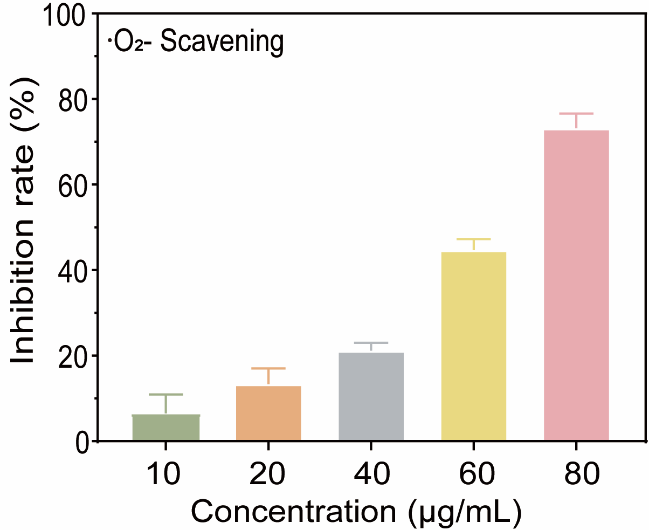


**Fig. S6** 🞄O_2_^-^ scavenging ability of 7CZ nanomedicines with different concentrations.


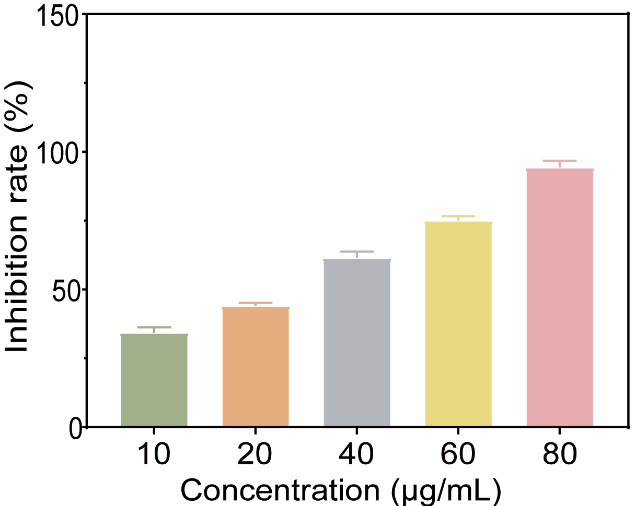


**Fig. S7** Qualitative analysis of ABTS radicals scavenging activity of 7CZ nanomedicines with different concentrations.


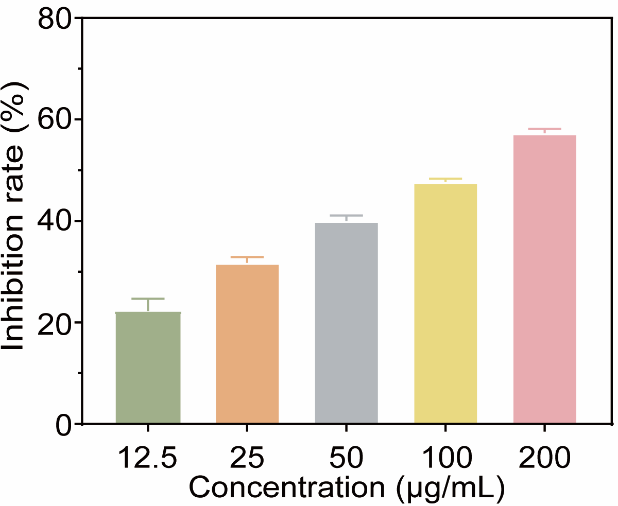


**Fig. S8** Qualitative analysis of DPPH radicals scavenging activity of 7CZ nanomedicines with different concentrations.


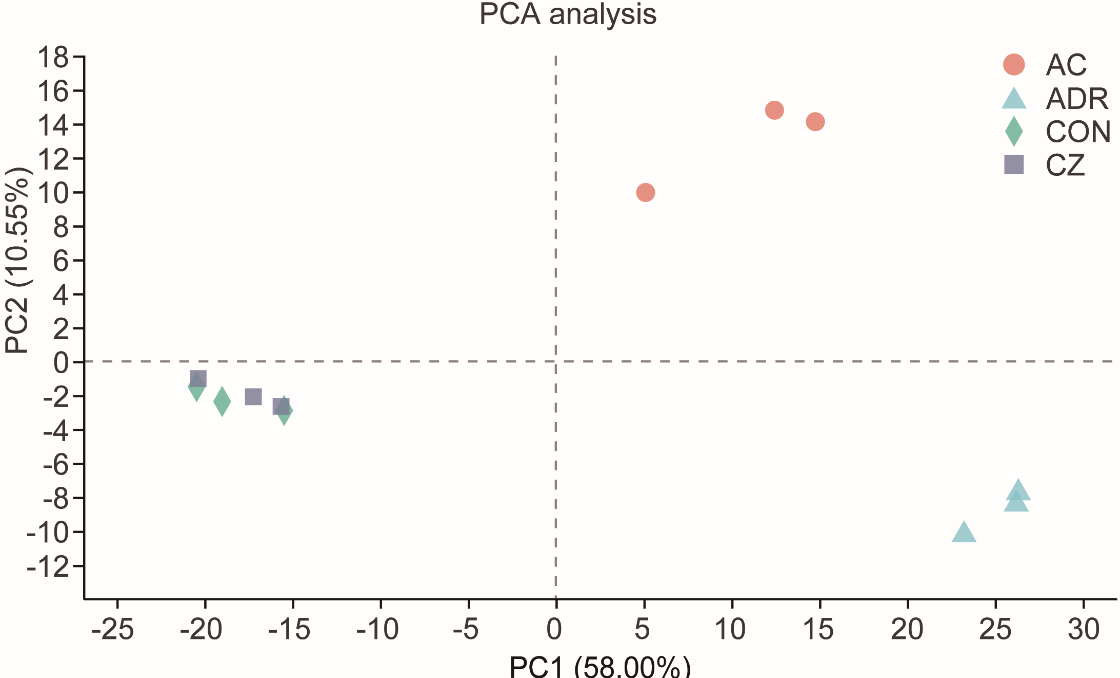


**Fig. S9** The no-guided principal component analysis (PCA) of different groups.


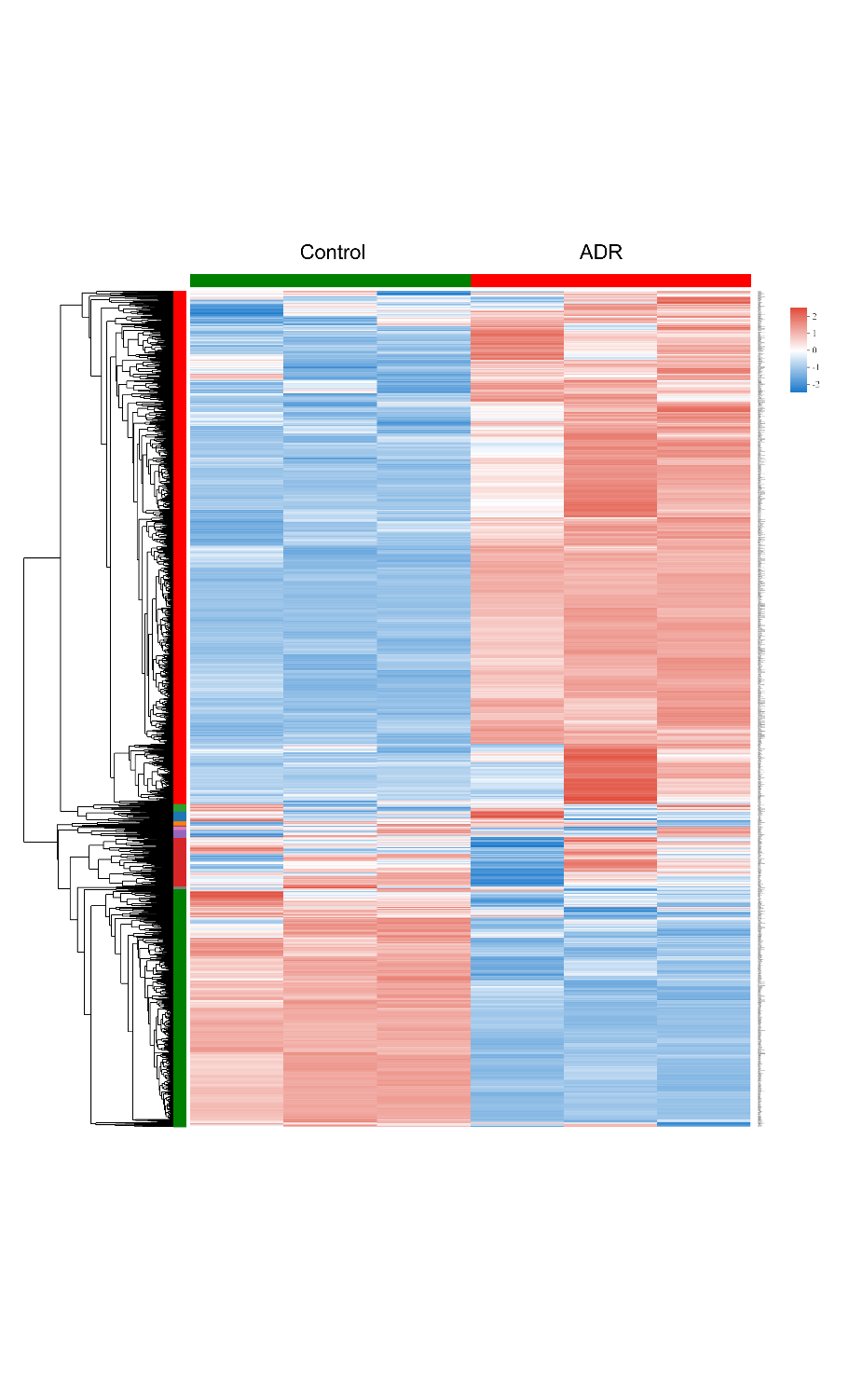
­

**Fig. S10** Heatmap of significant genes involved between control and ADR groups (fold change ≥2 and *P* < 0.05).
